# Electrometric and Electron Paramagnetic Resonance Measurements of a Difference in the Transmembrane Electrochemical Potential: Photosynthetic Subcellular Structures and Isolated Pigment–Protein Complexes

**DOI:** 10.3390/membranes13110866

**Published:** 2023-11-01

**Authors:** Alexey Yu. Semenov, Alexander N. Tikhonov

**Affiliations:** 1 A.N. Belozersky Institute of Physical-Chemical Biology, M.V. Lomonosov Moscow State University, 119991 Moscow, Russia; semenov@belozersky.msu.ru; 2 Faculty of Physics, M.V. Lomonosov Moscow State University, 119991 Moscow, Russia

**Keywords:** biomembranes, electrochemical potential, photosynthesis, electrometrical technique, electron paramagnetic resonance

## Abstract

A transmembrane difference in the electrochemical potentials of protons (Δμ_H+_) serves as a free energy intermediate in energy-transducing organelles of the living cell. The contributions of two components of the Δμ_H+_ (electrical, Δψ, and concentrational, ΔpH) to the overall Δμ_H+_ value depend on the nature and lipid composition of the energy-coupling membrane. In this review, we briefly consider several of the most common instrumental (electrometric and EPR) methods for numerical estimations of Δψ and ΔpH. In particular, the kinetics of the flash-induced electrometrical measurements of Δψ in bacterial chromatophores, isolated bacterial reaction centers, and Photosystems I and II of the oxygenic photosynthesis, as well as the use of pH-sensitive molecular indicators and kinetic data regarding pH-dependent electron transport in chloroplasts, have been reviewed. Further perspectives on the application of these methods to solve some fundamental and practical problems of membrane bioenergetics are discussed.

## 1. Introduction

The transmembrane difference in the electrochemical potential of protons plays a key role in energy transduction in photosynthetic organisms, mitochondria, and bacterial cells. The difference in proton potential across the energy-transducing membranes (Δμ_H+_, often termed as the proton motive force, *pmf*) serves as the driving force for the operation of the membrane-bound ATP synthase, which catalyzes the ATP formation from ADP and inorganic phosphate P_i_ [1,2,3,4,5,6,7]. Along with the energy role in membrane bioenergetics, the proton potential also participates in the regulation of electron transport processes and metabolic processes in living cells of different origins (for references, see [8,9,10,11,12,13]).

**Figure 1:**
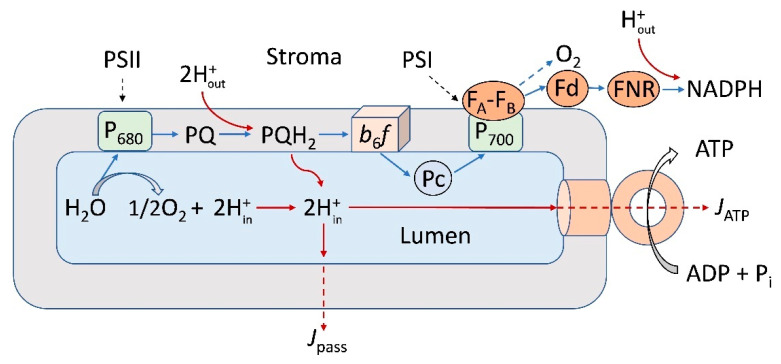
Scheme illustrating noncyclic electron transport (blue lines) and interaction between the electron transport complexes embedded in the thylakoid membrane (PSI, PSII, and the *b*_6_*f* complexes) and the mobile electron carriers (plastoquinone, PQ, plastocyanin, Pc, and ferredoxin, Fd). FNR—ferredoxin NADP reductase. P_700_ and P_680_ denote primary electron donors in PSI and PSII, respectively. The light-induced accumulation of hydrogen ions inside the chloroplast lumen and the transmembrane proton fluxes from the lumen to stroma are indicated by red lines. Arrows indicated by symbols *J*_ATP_ and *J*_pass_ symbolize the transmembrane proton fluxes coupled to operation of the membrane-bound ATP synthase complex and passive efflux of protons through the thylakoid membrane, respectively.

In energy-transducing membranes of photosynthetic organisms, mitochondria, and bacterial cells, generation of the *pmf* is associated with the functioning of electron transport chains (ETC). In photosynthetic systems of an oxygenic type (chloroplasts of high plants, algae, and cyanobacteria), the ETC includes two membrane-bound protein complexes, Photosystems I and II (PSI and PSII), which are interconnected via the cytochrome (Cyt) complex *b*_6_*f* and mobile electron carriers (plastoquinone and plastocyanin) [14]. Operating in tandem, PSI and PSII facilitate the transfer of electrons from the water molecule, oxidized by the water-splitting complex of PSII, to NADP^+^, the terminal electron acceptor of PSI (Figure 1). The light-induced electron transfer induces the translocation of protons into the *intra*-thylakoid volume (lumen) of chloroplasts, accompanied by the acidification of the lumen (pH_in_↓) and alkalization of the chloroplast stroma (pH_out_↑), causing the generation of *pmf*, which consists of two components: ΔpH (the *trans*-thylakoid pH difference) and Δψ (the *trans*-thylakoid difference of electric potentials). Along with the energetic role, *pmf* is involved in the feedback regulation of photosynthetic electron transport: the lumen acidification retards electron transfer between PSII and PSI at the level of plastoquinol (PQH_2_) oxidation by the Cyt *b*_6_*f* complex [15,16,17,18,19] and attenuates the PSII activity [9,10,20]. The alkalization of stroma activates the Calvin–Benson cycle (CBC) reactions [8,21]. In bacterial chromatophores, the cyclic electron transfer results in the translocation of protons inside chromatophores, generation of a transmembrane electric potential difference (Δψ), and ATP synthesis. 

The acclimation of photosynthetic apparatus to variable environmental conditions establishes an optimal balance between different metabolic processes in the plant cell. The effects of temperature are among the clue factors of the regulation of photosynthetic processes, because plants growing under a fluctuating temperature have no intrinsic mechanisms of temperature control, which are peculiar to animals (for references, see [22,23,24]). The adjustment of photosynthetic apparatus to variable environmental conditions should provide a proper balance between the electron and proton transport processes for generation of optimal *pmf*, used for efficient ATP synthesis. The light-induced regulation of *pmf* determines the photosynthetic efficiency and plant survival under the light fluctuations [23,24]. In chloroplasts, the composition of *pmf* (the proton-dependent concentration and electric components, ΔpH and Δψ) is controlled by several factors. The parsing of ΔpH and Δψ would change upon variations in temperature, light intensity, and nutrition [25,26]. In particular, the acclimation of photosynthetic apparatus to low temperatures induces the alteration in the membrane fatty acid composition, leading to the adjustment of the membrane fluidity and stabilization of proteins (for a review, see [24]). 

In this paper, we briefly discuss some biophysical methods used for measuring *pmf* in photosynthetic energy-transducing systems of different origin (photosynthetic bacteria, plant chloroplasts, and cyanobacteria). Our main attention has been focused on photosynthetic electron transport complexes (bacterial reaction centers, PSI, and PSII), chloroplasts, and chromatophores. We also consider the perspectives of the fabrication of artificial energy-transducing systems based on the use of photosynthetic energy-transducing complexes and analyze some fundamental aspects of their use in bioenergetics. 

## 2. The Proton Motive Force in Membrane Bioenergetics 

The *trans*membrane difference in the electrochemical potentials of protons (Δμ_H_^+^), expressed in electrical units, can be written as

Δμ_H_^+^ = Δψ + (2.3*RT*/*F*) ΔpH, (1)

where Δψ = ψ_in_ − ψ_out_ is the *trans*-thylakoid difference in electric potentials, ΔpH = pH_out_ − pH_in_ is the *trans*-membrane pH difference, *F* and *R* stand for Faraday’s and the universal gas constants, and *T* is temperature. Both components of Δμ_H+_, Δψ, and ΔpH are competent for driving ATP synthesis [3,4,27,28,29,30]. 

In chloroplasts, ΔpH is usually considered the basic contributor to Δμ_H_^+^ [10,31,32,33]. The energy stored in the form of ΔpH (ΔpH~1.0–1.5 or higher, which is equivalent to 60–90 mV) is sufficient to sustain efficient ATP synthesis, providing the stoichiometric ratio H^+^/ATP ≥ 4–5 (for references, see [34]). The steady-state values of Δψ are usually insignificant (Δψ < 20 mV). However, under certain experimental conditions, the deposition of Δψ to *pmf* cannot be ignored [10,31,32,33]. Although the ΔpH difference is usually considered the dominant component of Δμ_H+_, the relative contributions of Δψ and ΔpH to the overall value of Δμ_H+_ may vary, depending on the nature and lipid composition of the thylakoid membranes. The relatively low steady-state values of Δψ in chloroplasts can be explained by different reasons, e.g., due to peculiarities of the lipid composition and the ion-exchange processes in the thylakoid membranes. In particular, the light-induced pumping of protons into the thylakoid lumen is electrically compensated by the inward movements of Cl^−^ ions and the efflux of Mg^2+^ and K^+^ ions [35]. Although the secondary transport of ions would decrease Δψ, the *trans*-thylakoid pH difference (ΔpH) would build up, supporting the operation of the ATP synthase.

In the energy-transducing membranes of mitochondria and bacteria, in contrast to chloroplasts, the electric potential difference Δψ serves as the basic component of *pmf* [36,37]. One of the reasons for such a difference may be associated with the different compositions of the lipid membranes. The membranes of mitochondria and bacterial cells consist predominantly of phospholipids with polar heads [38,39,40,41,42]. This property may put restrictions on the membrane permeability for ions, suggesting that mitochondrial and bacterial membranes exhibit a rather high ability to maintain Δψ. Note that the ion-exchange processes may include the protonation of acidic groups of membrane proteins and lipids (A^−^ + H^+^ ↔ AH and/or B + H^+^ ↔ BH^+^). However, the protonation/deprotonation events of this kind would not change, per se, the electric charges on both sides of the energy-coupling membranes, and, therefore, the proton-binding reactions should not suppress the generation of Δψ. In the meantime, the processes associated with the proton exchange for cations (e.g., Mg^2+^ and/or K^+^) would reduce the activity of H^+^ ions in the bulk phases around energy-transducing membranes, thereby reducing ΔpH. Therefore, the mitochondrial energy-transducing membranes would be able to maintain a sufficiently high Δψ, while the ΔpH value will be reduced due to the H^+^ binding to the proton-accepting groups of membrane proteins and lipids. According to textbooks on bioenergetics [36,37], the dominant point of view is that in mitochondria, the Δψ values reach ~150–200 mV, while the characteristic pH difference between the outer (the intermembrane space) and internal (matrix) volumes does not exceed ΔpH~0.5–1.0 (about 30–60 mV).

The mechanisms of *pmf* regulation in chloroplasts is important for the productivity of plants in field crops [25]. Variability of *pmf* and relatively low steady-state values of Δψ in chloroplasts can be compensated for by the movements of other ions across the thylakoid membranes [35]. Also, we cannot ignore the “structure-function” reasons for the domination of ΔpH in the thylakoid membranes and the enhanced values of Δψ in mitochondria associated with different lipid bilayer compositions in chloroplasts and mitochondria. In chloroplasts, the electrically neutral galactolipids (monogalactosyldiacylglycerol (MGDG), and digalactosyldiacylglycerol (DGDG)) are the basic constituents of the lipid bilayer. Sulfoquinovosyldiacylglycerol (SQDG) and phospholipid phosphatydilglycerol (PG) are the minor components of the thylakoid membranes. In *Arabidopsis*, for example, MGDG constitutes about 52%, DGDG 26%, SQDG 6.6%, and PG 9.5% of the total membrane lipids [43,44,45]. MGDG and DGDG are electrically neutral lipids, while SQDG and PG are anionic lipids at physiological pH. One third of the total fractions of PG molecules in thylakoids of plants and cyanobacteria is integrated with protein complexes (PSI, PSII, and Cyt *b*_6_*f*), providing the structural organization of photosynthetic apparatus and optimal functioning of photosynthetic ETC [45]. Since the content of electrically neutral MGDG and DGDG is about 80% of the total number of the thylakoid membrane lipids, one may expect that the thylakoid membranes, containing predominantly uncharged galactolipids, reveal relatively high ion permeability, thereby providing a decrease in Δψ. In the meantime, the thylakoid membranes maintain a sufficiently high ΔpH, at the levels sufficient to facilitate the operation of the ATP synthase. 

Mitochondrial membranes are enriched with the lipids containing charged groups: phosphatidylcholine, phosphatidylethanolamine, phosphatidylinositol, phosphatidylserine, and phosphatidic acid. In yeasts and higher eukaryotes, phospholipids make up 75–95% of total mitochondrial membrane lipids [38,39,40,41,42]. This peculiarity of mitochondrial membranes would lead to more resistance for the passive proton transfer of ions, thereby supporting the maintenance of a sufficiently high Δψ. In favor of this explanation, we can compare earlier data on measuring the ion permeability of planar (artificial) lipid membranes fabricated from galactolipids and lecithin [46,47]. It was demonstrated that the specific ion conductivity of planar bilayers formed from thylakoid lipids was about one order of magnitude higher than that of lecithin bilayers [47]. Note that the permeability to protons of the phospholipid bilayers and the membranes formed from galactolipids (10^−8^–10^−4^ cm s^−1^) are significantly higher than the permeability to other monovalent cations. The permeability coefficient for protons is about six orders of magnitude higher than the permeability for K^+^ ions [48,49]. 

Concerning the molecular mechanisms of a fast proton flow through the energy-transducing membranes, there are good reasons to believe that the proton flow across the membranes could proceed by means of a jump along transient H^+^-bonded strands of water molecules inside the membranes (for references, see [50,51,52,53,54,55,56]). It has been suggested that protons cross the lipid bilayer by moving along the proton-conducting “water wires” supported by the oxygen atoms of lipids [55]. The weak acid groups of the membrane proteins may also contribute to the H^+^ flux across the lipid bilayer (for example, see [56]). 

Comparing the proton conductivities through the artificial lipid bilayers and the thylakoid membranes, the authors of work [47] concluded, however, that the passive permeability for protons cannot be determined solely by the properties of the galactolipid domains of the thylakoid membranes. They emphasize the essential role of ion channels in the regulation of Δψ generation in chloroplasts, rather than the passive efflux of protons through the bulk of the membrane lipids. The light-induced generation of Δψ would be restricted due to the ion-exchange fluxes. An increase in the lumen electric potential ψ_in_, induced by proton pumping into the lumen, would be neutralized (at least partly) by the influx of anions to the lumen and the outflow of cations from the lumen to stroma. It has been reported recently that the thylakoid membranes contain the VCCN1 protein, which manifests the Cl^−^ channel activity [57]. The data obtained suggest that VCCN1 is involved in dissipating the Δψ component at high-light irradiation. The establishment of the electric potential difference Δψ across the thylakoid membranes is a dynamic process. The thylakoid membranes of *Arabidopsis* contain other ion transport proteins (the K^+^ efflux antiporters KEA1, KEA2, and KEA3 [58,59]), which are involved in osmo- and pH regulation in chloroplasts. The ion fluxes through the K^+^ channels would reduce Δψ, shifting the *pmf* value toward ΔpH. The putative K^+^/H^+^ antiporter KEA3 stimulates the Δψ generation. The importance of the mechanisms of the down-regulation of Δψ is supported by the finding that a sudden increase in Δψ may cause damage to PSI and PSII [60,61,62]. It is important to note that although the steady-state values of Δψ in illuminated chloroplasts may be relatively small, the ΔpH difference, induced by proton pumping, would maintain ΔpH at sufficiently high levels, thereby facilitating efficient ATP synthesis.

The energy-transducing membranes of cyanobacteria are of peculiar interest for membrane bioenergetics: these membranes contain both the photosynthetic and respiratory components of ETC, incorporated into the same membrane [63,64]. Cyanobacterial membranes are characterized by a higher content of anionic lipids [65] compared with thylakoid membranes of plant chloroplasts. Therefore, there are good reasons to expect that the energy-transducing membranes of cyanobacteria can maintain a sufficiently high level of Δψ when used as the energy supply for driving the ATP synthase. 

## 3. Overview of the Methods for Measuring Δψ and ΔpH in Biomembranes 

Bearing in mind the importance of *pmf* as a free energy intermediate, it is not surprising that a great number of works in the field of membrane bioenergetics have been devoted to the determination of *pmf* values and numerical parsing of Δψ and ΔpH in energy-transducing biological systems (for references, see [3,4,27,28,29,30,31,32,33,34]). In this section, we present a brief overview of the widely used methods for monitoring the Δψ and ΔpH components of *pmf* by using electrometrical, optical, and electron paramagnetic resonance (EPR) methods. Our attention is focused on photosynthetic energy-transducing systems. Besides the scientific importance of photosynthetic membranes, these systems attract scientists because they allow us to study the fast kinetics of charge transfer processes due to the convenient initiation of electron transport by short light pulses. In order to illustrate the methodological aspects of measuring *pmf* via biophysical (instrumental) methods, we briefly consider the approaches for determination of Δψ and ΔpH that have been used in our scientific groups.

### 3.1. Synthetic Penetrating Ions

A method based on using the membrane-penetrating synthetic ions was developed in the laboratories of E.A. Liberman and V.P. Skulachev [66]. According to this method, an artificial phospholipid membrane (ALM) was used to separate two compartments of the measuring cell, serving as a selective electrode for the synthetic penetrating anions, such as phenyldicarbaundecaborane and tetraphenylborate. The uptake of these anions by submitochondrial particles, chromatophores, or bacteriorhodopsin-containing proteoliposomes, initiated by the addition of substrates or illumination, caused a decrease in the anion concentration inside the compartment of the experimental cell, where the vesicles were added and induced the appearance of a potential difference across the ALM [67,68]. The employment of the different membrane porous filters as the frames for ALM expanded the application of the method by using penetrating cations. This allowed the researchers to monitor the Δψ generation in vesicles with a negative potential inside, like mitochondria, bacteria, and some types of proteoliposomes [69].

### 3.2. Electrochromic Band Shift of Carotenoid Spectrum 

A method based on the detection of the carotenoid absorption changes due to electrochromic shifts in their spectra in response to membrane potential generation was commonly used for Δψ measurements in photosynthetic organisms [70]. The amplitude of the carotenoid absorption changes induced by an electric field was shown to be a linear function of the transmembrane potential [70,71]. This approach has been widely used in studies on electrogenic reactions in the chromatophores of purple bacteria and the thylakoids of chloroplasts. The method, however, was ineffective in studies on isolated pigment–protein complexes in model systems and cyanobacterial cells. In addition, the carotenoid shift was characterized by a relatively low signal-to-noise ratio, which required signal averaging of the kinetic traces. It should be noted that, later, Joliot and Joliot [72] developed a “kinetic” spectrophotometer that achieves a higher signal-to-noise ratio by utilizing differential optics and a series of relatively intense light pulses from a flash lamp or laser instead of a continuous beam to probe the sample. Nevertheless, the data obtained by measuring the carotenoid band shifts for a quantitative evaluation of Δψ generation should be considered with caution, because they reflect the appearance and decay of local charges on redox cofactors in the course of electron transfer and depend on the relative arrangement of the charged redox cofactors and carotenoid molecules. 

Recently, using PSI complexes from cyanobacteria and monitoring the recombination kinetics from the reduced plastoquinone acceptors A_1A_^−^ and A_1B_^−^ in the symmetrical branches of redox cofactors to the photo-oxidized primary electron donor P_700_^+^, we came to the conclusion that the carotenoid molecules located in the vicinity of A_1A_ and A_1B_ revealed different sensitivies to the negative charges on A_1A_^−^ and A_1B_^−^ [73]. It was shown that the amplitude of the carotenoid band shift measured at 480 nm It was shown that the amplitude of the carotenoid bandshift measured at 480 nm and attributed to recombination from A_1A_^−^ should be multiplied by a factor of 2.3 to obtain the true relative contribution of electron transfer from P_700_^+^ to A_1A_. This correction is reasonable given that the arrangement of carotenoid molecules within the protein in relation to A_1A_ and A_1B_ is different. 

### 3.3. The Direct Electrometrical Method of Δψ Measurements

**Figure 2:**
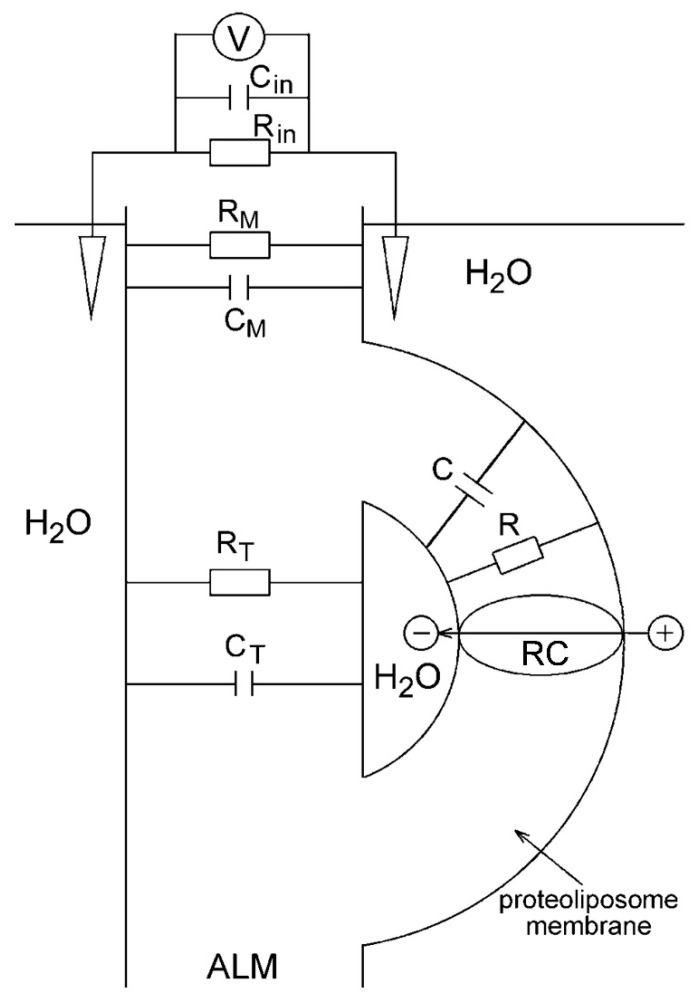
A schematic diagram of the direct electrometrical measurements in the reaction center (RC) containing proteoliposome–ALM system. *C*_P_, *R*_P_, *C*_M_, *R*_M_, *C*_F_, *R*_F_—the electric capacitances (*C*) and resistances (*R*) of the proteoliposome membrane (*C*_P_, *R*_P_), ALM (*C*_M_, *R*_M_), and a fusion region of the proteoliposome and ALM (*C*_F_, *R*_F_), respectively; V—electrometric voltmeter or operational amplifier with high internal resistance *R*_in_ and low internal capacitance *C*_in_. Adapted from [75], with permission.

The direct electrometrical method was first used in studies on electrogenesis in the membranes of proteoliposomes containing bacteriorhodopsin, mitochondrial Cyt *c* oxidase, and H^+^-ATPase [74]. The method is based on the association of closed protein-lipid–vesicles (proteoliposomes or bacterial chromatophores) with an ALM and on Δψ measurements with Ag/AgCl macroelectrodes immersed in an electrolyte solution on both sides of the ALM and connected to an electrometric voltmeter (or to a broad-band-operating amplifier when detecting fast kinetics). The incubation of vesicles for ~1 h in the presence of the divalent cations (Ca^2+^or Mg^2+^) was required to neutralize negative charges at the surfaces of the ALM and vesicle membranes and resulted in the association of vesicles with the ALM. The ALM-bound vesicles retained their inner aqueous phase (see simplified scheme of measurements in Figure 2 [75]). 

In early experiments with continuous illumination of the system, ALM represented a thick membrane formed from phosphatidylcholine solution in n-decane [74]. An upgrade of the measuring system included the use of membrane porous filters such as the ALM frame [69]. However, the low electric capacitance of a thick ALM (*C*_m_), which was comparable to the input capacitance *C*_in_ of the operational amplifier (~5 pF), resulted in distortion of the kinetics of the Δψ rise, induced by short laser pulses. A thin but sufficiently stable collodion (nitrocellulose) film impregnated with phospholipid solution with much higher capacitance *C*_m_ was used in our experiments for detecting fast kinetics of Δψ generation [76]. 

The resistance of the collodion membrane *R*_m_ was significantly lower than internal resistance *R*_in_ of the amplifier, whereas its capacitance (*C*_m_~5000 pF) was larger by three orders of magnitude than the internal capacitance *C*_in_ of the operational amplifier. The use of the phospholipid-impregnated collodion film as an ALM provided an opportunity to detect undistorted electric signals with a time resolution of ~200 ns.

### 3.4. The Use of pH-Sensitive Molecular Probes 

For many years, the question “How acidic is the lumen?” was the focus of bioenergetics [77]. It has become the textbook view that the *trans*-thylakoid pH difference (ΔpH = pH_out_ − pH_in_) provides the main contribution to *pmf* in chloroplasts [36,37]. However, the data available from the literature are often ambiguous, supporting either a moderate acidification of the *intra*-thylakoid volume (pH_in_~5.8–6.5) or strongly acidic lumen (pH_in_ < 5). According to most earlier works, ΔpH reaches up to ΔpH > 3–3.5 (for references, see [17]). In this subsection, we consider the use of pH-sensitive molecular probes for measuring ΔpH in chloroplasts. Our particular interest will concern the advantages of using the electron paramagnetic resonance (EPR) technique for noninvasive determination of ΔpH in chloroplasts in situ. 

There are several kinds of molecular indicators used for measuring ΔpH; for example, the fluorescence dyes, the optical absorption, and fluorescence spectra, which are sensitive to pH changes (e.g., neutral red, NR). In particular, pH-sensitive dyes were often used in earlier works on tracking the proton release inside the thylakoids [78,79]. The basic problem of using pH-sensitive dyes is that most of the widely used molecular indicators bind to the thylakoid membranes, leading to overestimations of ΔpH by 1.0–1.5 pH units [80,81]. According to Junge et al. [78], NR is one of the “clean” indicators of the *intra*-thylakoid pH (pH_in_), with which spectral changes show no response to other light-induced events in chloroplasts. Another problem is that the absorption and fluorescence spectra of the pH-sensitive dyes often overlap with the spectra of photosynthetic pigments, thereby complicating ΔpH measurements. Also, determinations of the inner volume of thylakoids (*V*_in_), which is necessary for estimations of ΔpH from partitioning the pH indicators between the inner and outer volumes of a suspension, can be obscured by osmotic effects. Thus, it is not surprising that the records of ΔpH in chloroplasts are often ambiguous (see, for comparison, the works [82,83,84,85]). 

The electron paramagnetic resonance (EPR) technique helps overcome the above-mentioned methodological problems. In general, there are two basic approaches to measuring pH_in_ by EPR: (1) the use of pH-sensitive molecular indicators (paramagnetic spin probes, for references, see [34,86,87,88,89]), and (2) the determination of pH_in_ based on measuring the rates of pH-dependent steps of electron transport in chloroplasts [90,91,92]. These approaches have been used in our previous works; some of the examples illustrating these methods are presented below.

#### 3.4.1. Partitioning the Indicator Molecules between the Lumen and External Volume

In earlier works, ΔpH values in chloroplasts were derived from partitioning the penetrating amine derivatives between different compartments separated by the thylakoid membrane [83,84,85]. Paramagnetic derivatives of amines (the nitroxide radicals containing the amine groups) are the most suitable ΔpH indicators of this type, which accumulate inside the thylakoids in response to the lumen acidification in the result of the protonation of spin probe molecules. Similar to other amines used to measure ΔpH [86,87,88,89], paramagnetic derivatives of amines are taken up by thylakoids in response to ΔpH generation.

**Figure 3:**
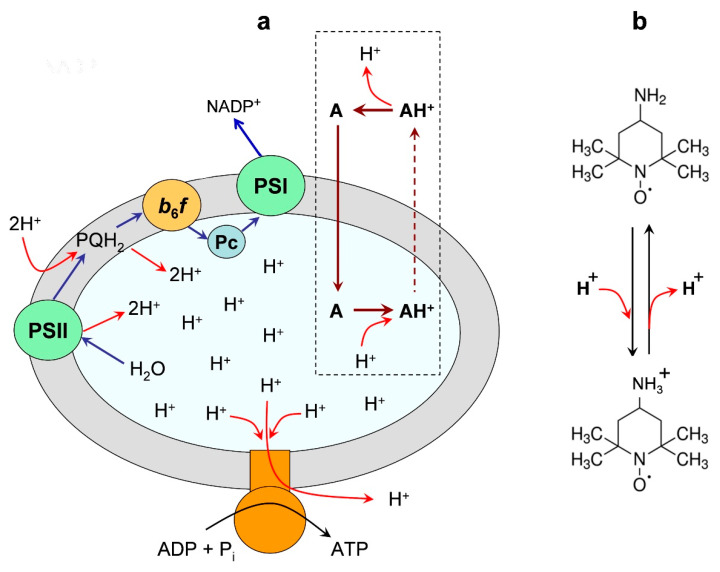
Panel (**a**) is an illustration of the light-induced redistribution of amine-based pH indicator Tempamine (4-amino-2,2,6,6-tetramethylpiperidine-1-oxyl, TA) between the outer space and the internal volume (lumen) of a chloroplast thylakoid. Panel (**b**) shows the deprotonated and protonated forms of the spin probe TA. Two different vertical arrows (continuous and dashed lines) symbolize the high and low permeability of the neutral and charged forms of TA through the membrane. Modified Figure 3 from [88], with permission.

Figure 3 demonstrates a distribution of a spin probe Tempamine (4-amino-2,2,6,6-tetramethylpiperidine-1-oxyl, TA) between the chloroplast lumen and the outer bulk phase. One of the advantages of using TA as the ΔpH indicator is that TA molecules do not bind to the thylakoid membranes. An experimenter can calculate ΔpH as:

ΔpH = log([H^+^]_in_/[H^+^]_out_) ≈ (*A*_in_/*V*_in_)/(*A*_out_/*V*_out_), (2)

where *V*_in_ and *V*_out_ stand for the relative volumes of the lumen and the outer space [83,84,85]. An experimenter can distinguish the EPR spectra of the indicator molecules localized inside and outside the thylakoids. This is possible because the EPR signals from spin probes accumulated in the osmotic volume of thylakoids (lumen) would reveal EPR spectra that are different from the spectra of a spin probe localized in the water bulk phase outside the thylakoids (for references, see [88,89]). The internal concentration of a spin probe (*A*_in_) and the internal volume of thylakoids (*V*_in_) can be found by measuring a number of TA molecules located in the bulk phase of the lumen. The observation of the EPR spectra of spin probes localized in the lumen can be performed by quenching the EPR signal of the external spin probe molecules, using chemically inactive paramagnetic agents (e.g., chromium oxalate molecules), which do not penetrate into the lumen (for technical details, see explanations in [34]). Thus, by measuring the amounts of indicator molecules inside and outside the vesicles (*A*_in_ and *A*_out_), and determining the ratio *V*_in_/*V*_out_, we can calculate ΔpH via Equation (2).

A sophisticated method for measuring ΔpH with TA, which does not need the knowledge of the internal volume *V*_in_, has been developed in our work [89]. This method is based on the threshold effect of the concentration-dependent broadening of the EPR signal from TA molecules accumulating in the thylakoid lumen. An increase in the concentration of TA molecules inside the lumen ([TA]_in_) leads to the broadening of their EPR spectra due to spin–spin interactions. The broadening effect reveals itself when [TA]_in_ overcomes the threshold level [TA]_th_ ≈ 2–2.5 mM. Measuring the light-induced responses of the EPR spectra at different concentrations of TA in the chloroplast suspension, we were able to find the concentration of TA outside the thylakoids, [TA]_out_, which corresponded to the internal concentration [TA]_in_ = [TA]_th_. Then, using the formula ΔpH ≈ log_10_([TA]_in_/[TA]_out_), we can calculate ΔpH. It is important to stress that this approach allowed us to avoid difficulties that might be caused by osmotic events (e.g., the light-induced swelling of thylakoids) [89]. This is because we determine the concentrations of TA inside and outside the thylakoids directly, on the basis of the EPR broadening effect, avoiding the measurements of a number of spin probe molecules, the lumen, and its volume. 

#### 3.4.2. The Light-Induced Changes in the EPR Spectra of pH-Sensitive Spin Probes 

This approach to the determination of pH_in_ relies on measuring the EPR spectra of specially tailored nitroxide radicals, the EPR signals of which are sensitive to changes in pH [34,93]. Loading the thylakoid lumen with the pH-dependent spin probes, an experimenter can determine pH_in_ from the EPR spectra of these molecules. Among different kinds of pH probes, imidazolidine and imidazoline derivatives of nitroxide radicals are the most suitable pH indicators for measuring pH_in_ in chloroplasts [34]. 

**Figure 4:**
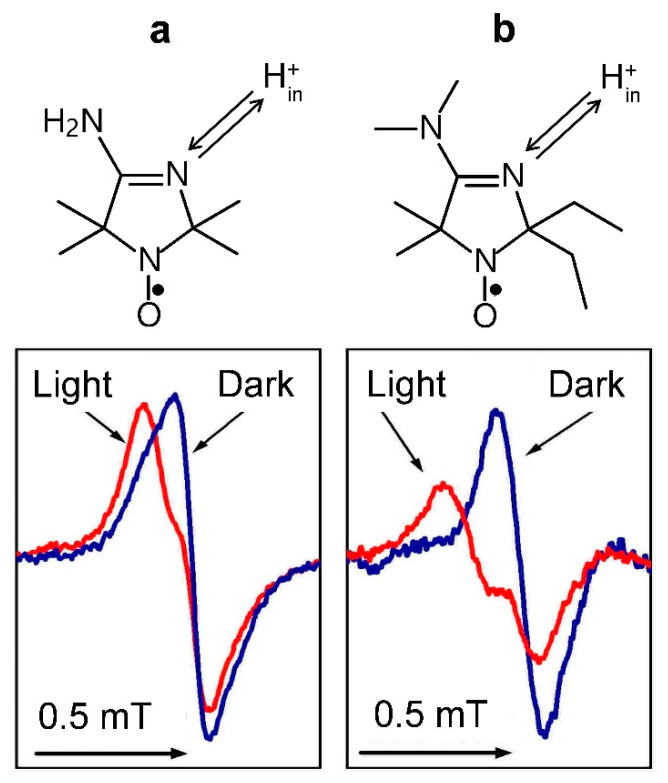
The high-field components of the EPR spectra of pH-sensitive nitroxide radicals 4-amino-2,2,5,5-tetramethyl-2,5-dihydro-1H-imidazol-1-oxyl (**a**) and 4-dimethylamino-2,2-diethyl-5,5-dimethyl-2,5-dihydro-1H-imidazol-1-oxyl (**b**) localized inside bean thylakoids. In both cases, illumination of chloroplasts by white light leads to the low-field shifts of the lines due to the acidification of the radical surroundings. Arrows at the top symbolize the protonation/deprotonation of the N atoms of the spin probes. Modified fragments of Figure 3 (panels **b** and **c**) from [34], with permission.

Figure 4 illustrates how the light-induced acidification of the lumen of isolated bean chloroplasts induces changes in the line shapes of two different spin probe molecules localized inside the thylakoids. We could extract these EPR signals using the line-broadening technique based on the addition of the membrane’s impermeable chromium oxalate (CrOx) molecules to the chloroplast suspension. Paramagnetic CrOx molecules caused significant broadening of the EPR signal of spin probe molecules dissolved in the aqueous bulk phase outside the thylakoids but did not influence the EPR signal of spin probe molecules localized in the lumen. Therefore, we were able to monitor the light-induced changes in pH_in_. Having an appropriate calibration curve (the pH dependence of the EPR spectrum parameters of spin probes), we could determine the light-induced changes in the lumen pH_in_ (for experimental details, see [34]). According to our study, in isolated bean chloroplasts functioning in the state of photosynthetic control (without the net proton efflux from the lumen to stroma via the ATP synthase), we determined pH_in_ ≈ 5.4–5.7 in illuminated bean chloroplasts suspended at pH_out_ = 7.8–8.0. In chloroplasts functioning under photophosphorylation conditions, pH_in_ decreased to pH_in_ ≈ 6.2. Such a decrease in pH_in_ is consistent with a point of view that under steady-state conditions, the proton gradient ΔpH is the main contributor to the proton motive force driving the operation of ATP synthesis. 

The structural features of CF_0_CF_1_-type ATP synthase put some demands on pH_out_ and pH_in_, the values of which ensure the enzyme operation in the ATP synthesis mode. Rotation of the *c*_n_-ring of the membrane-bound protein ensemble CF_0_ in the direction that is necessary for ATP synthesis would proceed if pH_in_ ≤ p*K*_A_ ≤ pH_out_. Here, p*K*_A_ is the effective p*K* value of crucial proton-accepting groups of the enzyme involved in the H^+^ translocation through the ATP synthase [17]. If these conditions hold true, then the reiterating protonation/deprotonation events in the CF_0_CF_1_ complex would ensure the ATP formation. The thermodynamic requirement for ATP synthesis is given by the relationship Δ*G*_ATP_ ≤ *m*∙Δμ_H_^+^, where Δ*G*_ATP_ is the standard Gibbs free energy for ATP formation from ADP and inorganic phosphate (P_i_), and *m* = H^+^/ATP is the number of protons pumped through the ATP synthase per ATP formed [4,5,6,7]. One complete turn of the ring *c*_n_ yields the formation of three ATP molecules. The stoichiometric ratio *m* = H^+^/ATP is determined by a number *n* of *c* subunits (*m* = *n*/3) that compose the *c*_n_ ring of the membrane-buried fragment of the ATP synthase. The number of *c* subunits per one ATP synthase varies between species from 8 to 17 [94,95,96,97,98]. The free energy Δ*G*_ATP_ required for ATP synthesis in chloroplasts depends on the metabolic status of the system. In chloroplasts functioning under physiological conditions, this value is about 50 kJ/mol (the energy equivalent of *pmf* is about 530 eV [96]). The apparent H^+^/ATP ratio in chloroplasts (*m* ≈ 4.7) allows photosynthetic organisms to produce ATP at relatively low *pmf* [98]. Summing up the energies of *m* protons translocated through the CF_o_ complex, and assuming that under physiological conditions, *pmf* is stored mainly in the form of ΔpH, we obtain that the energy of several protons (*m* = 4–5) should be enough to sustain the steady-state ATP synthesis if ΔpH = 1.8 (at pH_out_ = 8) [34].

#### 3.4.3. Surface Potential Measurements

In addition to measuring pH_in_ with pH-sensitive spin probes, one can use amphiphilic spin probes for detection of the light-induced changes in the thylakoid membrane surface potential (Δψ_s_). This approach is based on the fact that charged amphiphilic molecules, which have paramagnetic fragments (nitroxide radicals), are distributed between the lipid moiety of the membrane and the water bulk phase [99,100,101]. EPR spectra of amphiphilic nitroxide radicals are sensitive to the polarity of their local surroundings. The light-induced changes in the membrane potential would lead to the redistribution of spin probe molecules between the membrane moiety and the water bulk phase. Positively charged probe molecules get involved in the lipid membrane when the membrane surface acquires a negative charge; otherwise, negatively charged probe molecules are expelled from the membrane. Thus, monitoring the EPR spectra of appropriate amphiphilic spin probes, an experimenter could determine the light-induced changes in the surface potential and calculate Δψ_s_ values. In bean chloroplasts, the light-induced decrease in the surface potential ψ_s_ was estimated as Δψ_s_ ≈ −15 mV [101].

### 3.5. pH-Dependent Kinetics of Photosynthetic Electron Transport in Chloroplasts 

Independently of using the spin probe techniques described above, an experimenter can determine pH_in_ via the so-called “kinetic” method, which relies on the study of the rates of electron transport processes controlled by the *intra*-thylakoid pH_in_ [14,15,17,90,91,92]. In chloroplasts, the pH-dependent stage of electron transfer between PSII and PSI is associated with the electron flow from the plastoquinol pool (PQH_2_) to oxidized reaction centers P_700_^+^ (via the Cyt *b*_6_*f* complex and plastocyanin) [15,16,17,18,19]. Measuring the rate of electron transport and having an appropriate calibration dependence (the rate of electron transfer vs. pH_in_), an experimenter can determine pH_in_ in different metabolic states of chloroplasts. We can say that the electron transport chain serves as a “local pH-meter”, which measures pH_in_ in the *intra*-thylakoid domains adjoining the Cyt *b*_6_*f* complex. An important advantage of this method is that it provides a noninvasive instrument for determination of pH_in_: an experimenter does not need to introduce into the system special pH-indicating molecules that might disturb the operation of the chloroplast ETC. Under experimental conditions, when the ATP synthase does not work, this method yields a ΔpH values close to that determined with spin probes (ΔpH ≈ 2.0–2.5). Under the photophosphorylation conditions, when the leak of protons through the ATP synthase complexes occurs efficiently, the results of pH_in_ measurements via spin probe and “kinetic” methods may be different. The “kinetic” method often yields less significant ΔpH values (ΔpH~1.0–1.8) compared with spin probe methods. According to [34,91,102], these ΔpH can ensure ATP synthesis from ADP and orthophosphate P_i_.

### 3.6. Lateral Heterogeniety of ΔpH

We can explain the inconsistence in the results of ΔpH measurements through different methods (spin probe techniques and kinetic data), taking into account the lateral heterogeneity of the chloroplast lamellar system. The uneven distribution of electron transport and ATP synthase complexes paves the way for different ΔpH values established across the membranes of grana- and stroma-exposed thylakoids. Significant parts of the proton pumps and proton sinks are located in different chloroplast domains: (a) most of the protons released from H_2_O molecules oxidized in PSII enter the lumen of granal thylakoids; (b) the proton leak from the stromal thylakoids occurs through the ATP synthase complexes localized predominantly in stroma-exposed thylakoids. Our argument in favor of the uneven distribution of ΔpH was based on the comparison of the results of ΔpH measurements via different methods. A similar notion about the heterogeneous lateral profile of pH_in_ was reached by Haraux, de Kouchkovsky, and collaborators, who investigated the isotopic exchange effects (H_2_O/D_2_O substitution) on energy-coupled processes in chloroplasts [103,104]. 

**Figure 5:**
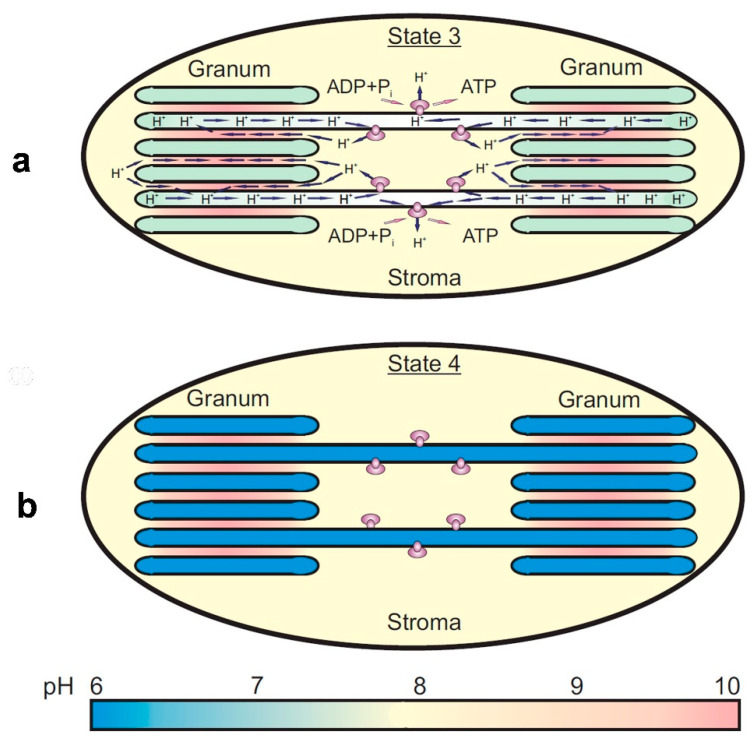
Schematic representation of the nonuniform (**a**) and uniform (**b**) modes of proton partitioning inside the granal and stromal domains of laterally heterogeneous chloroplasts (modified 515 Figure 11 from [102], with permission). Significant alkalization of the gap between appressed thylakoids of grana can be explained by the proton uptake on the acceptor side of PSII and slow long-range diffusion of protons inside the gap. Short arrows indicate the lateral and *trans*-thylakoid pathways of the proton transfer associated with the operation of ATP synthases.

A sketch, illustrating the appearance of the nonuniform lateral profile of ΔpH, is shown in Figure 5. The leakage of protons from the lumen through the ATP synthase complexes of stroma-exposed thylakoids would cause a less significant decrease in ΔpH established across the membranes of the stromal thylakoids compared with ΔpH established across the membranes of granal thylakoids (Figure 5a). A hindrance to the long-range lateral diffusion of H^+^ ions from the lumen of granal thylakoids to the ATP synthases would provide the establishment of the lateral gradient of protons along the chloroplast lamellas. If the ATP synthase complexes are inactive, a proton equilibrium between the lumens of grana and stromal thylakoids would be established (Figure 5b). A mathematical model described in [93] supports the notion that the restrictions to the long-range obstructed diffusion of protons inside the lumen could influence the lateral profiles of ΔpH along the thylakoid membranes. The model predicts the establishment of nonuniform lateral profiles of ΔpH under the photophosphorylation conditions and significant alkalization of the *inter*-thylakoid gap. Theoretical analysis of the problem suggests that the hindrance to the long-range diffusion of protons inside the gap is due to significant alkalization of the *inter*-thylakoid gap (for details, see [102] and references therein).

Panel **a** in Figure 5 illustrates how the obstructed long-range diffusion of H^+^ ions from the lumen of granal thylakoids to the lumen of stroma-exposed thylakoids (enriched with the ATP synthases) hinders the establishment of the proton equilibrium between these thylakoids under photophosphorylation conditions. In this case, ΔpH across the membranes of stroma-exposed thylakoids is smaller than that in grana-exposed thylakoids, without randomization of protons between the thylakoids of both kinds. A fast leakage of protons from the lumen of stroma-exposed thylakoids through the ATP synthases would restrict a too strong acidification of the lumen of stromal thylakoids. Due to the slowing down of the long-range diffusion of protons inside the lumen, the lateral gradient of pH along the chloroplast lamellas will be established. 

Panel **b** in Figure 5 symbolizes the uniform distribution of protons inside the granal and stromal thylakoids when the ATP synthase complexes do not work. In this case, randomization of protons inside the thylakoids of both kinds will take place. 

## 4. Basic Results of *pmf* Measurements

### 4.1. Direct Electrometrical Measurements 

Initially, the direct electrometrical method was used for the demonstration of electrogenic activity of different membrane proteins participating in the transmembrane charge transfer, including bacteriorhodopsin, mitochondrial Cyt *c* oxidase, H^+^-ATPase [66], and bacterial reaction centers (bRC) incorporated into proteoliposome membranes. Besides, using natural closed vesicles (chromatophores) the electrogenic charge transfer due to the functioning of the photosynthetic electron transfer chain, inorganic pyrophosphatase, and H^+^-ATPase have been shown [105]. 

These results directly demonstrated the existence of the membrane proteins generating the transmembrane electric potential difference. This finding strongly supported one of the most important predictions of Peter Mitchell’s chemiosmotic theory, for which he was awarded the Nobel Prize in Chemistry in 1978. 

An electrometric technique was used later to study the kinetics of the electrogenic events accompanying charge transfer in bRC and two photosystems, PSI and PSII, from cyanobacteria and spinach (for details of 3D crystal structures of PSI and PSII, see [106,107] and references therein). It has been shown that in all types of RCs, the fast electrogenic reactions occur with a lifetime shorter than 200 ns between the chlorophyll (Chl) or bacteriochlorophyll (BChl) special pair dimer and quinone acceptors via monomeric (B)Chl and (in the case of PSII and bRC) via pheophytin (bacteriopheophytin). The rise time of the fast kinetic phase was limited by the electrical parameters of the operational amplifier and electrodes. In the case of PSII, electrogenic reactions on the donor side included the reduction of the (B)Chl dimer from the water soluble Cyt *c* or plastocyanin in the case of PSI and bRC (characteristic time τ was in the range of 1–100 µs) and the sequential reduction from redox-active tyrosine Y_z_ (τ~30 ns) and the water-oxidizing complex (τ~50 µs). Electrogenesis on the acceptor side included protonation of the double-reduced secondary quinone in bRC and PSII (τ~200 µs) and electron transfer from the quinone(s) to the 4Fe4S clusters F_X_, F_A,_ and F_B_ (τ~200 ns) and further to ferredoxin (τ in the range of 1–100 µs). These measurements, together with potentiometric titration of certain kinetic components, allowed us to identify the nature of all electron donors and acceptors as well as the sequence of charge transfer reactions. Moreover, the comparison of the relative contribution of charge transfer reactions to the overall electrogenesis with the projection of the distance vector between redox cofactors to the normal membrane allowed us to estimate the pattern of the intraprotein dielectric profile in bRC, PSI, and PSII [108]. 

**Figure 6:**
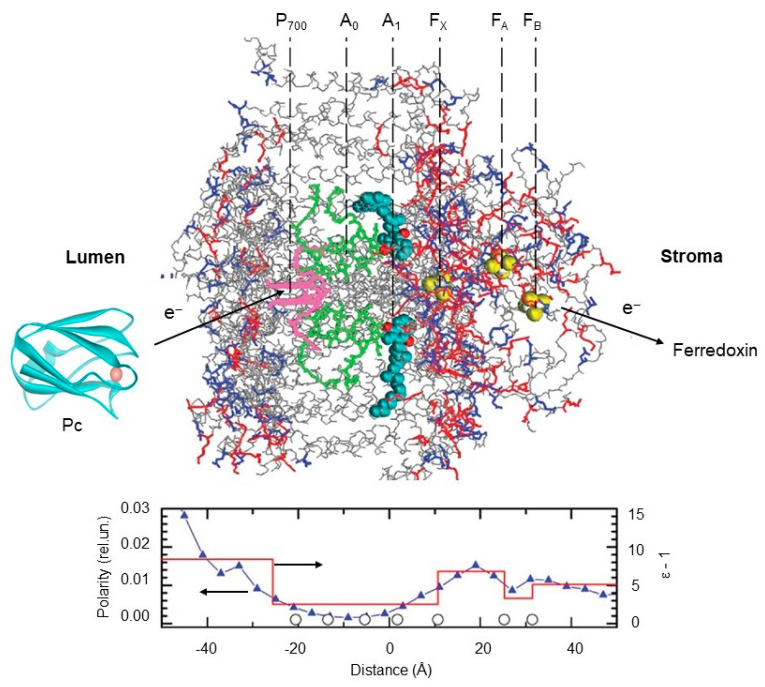
Distribution of polarity and dielectric permittivity along PSI complex. The 3D structure of the PSI complex was taken from [108]. Top panel shows three-dimensional (3D) structures of cyanobacterial PSI at 2.5 angstroms resolution (PDB code: 1jb0), spinach plastocyanin, Pc (PDB code: 1ag6), and distribution of oxygen and nitrogen atoms of polar amino acid side chains (red) and crystallographic water (blue) in relation to the redox cofactors in PSI complex. Pc structure was produced using Accelrys DV visualizer software package (http://www.accelrys.com). Bottom panel: distribution of the average protein polarity (blue triangles) and the effective dielectric permittivity ε (red line) across the membrane. Open circles along the ‘‘distance’’ axis mark the places of cofactors localization (see inscriptions above the protein structure). Polarity was defined as the number of polar atoms within a 4 Å thin layer parallel to the membrane plane divided by the whole number of atoms within this layer. Dielectric permittivity is determined from the electrometric experiments. The 3D structure of the PSI complex and the protein 577 polarity profile are adopted from [108], with permission.

The electrometric data revealed a notable variation in the dielectric properties of PSI in the normal direction to the membrane plane: the polarity was found to be lower in the middle of the membrane than at its edges [108]. Figure 6 shows the profiles of polarity and dielectric permittivity (ε) along the PSI complex. For the hydrophobic core of the complex, a low value of ε equal to 3.5 was accepted. The pattern of dielectric permittivity obtained by the electrometric data correlates strongly with the polarity of the PSI molecular structure. The microscopic polarity has a wide minimum in the middle of the membrane around the accessory chlorophyll A_0_ (approximately between the chlorophyll dimer P_700_ and the phylloquinone molecules A_1_) and gradually increases toward both edges of the complex. In the acceptor part of the PSI, the pattern of polarity reveals a maximum between 4Fe4S clusters F_X_ and F_A_ and a local minimum between F_A_ and F_B_. The profile of protein polarity is in qualitative agreement with the dielectric profile (red line in Figure 6, bottom panel) obtained via electrometric measurements. 

**Figure 7:**
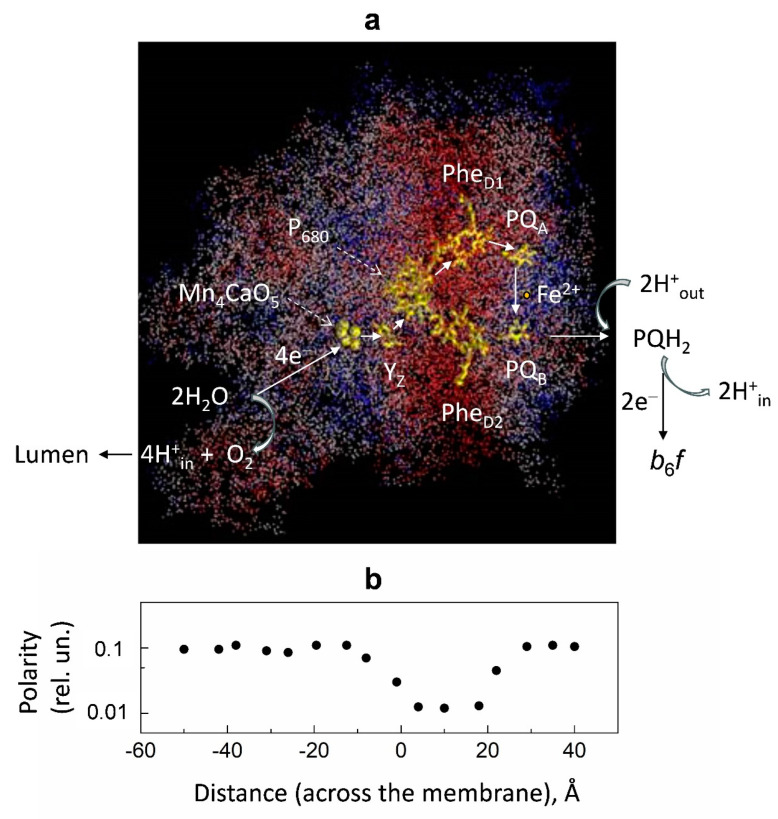
Panel (**a**): Distribution of relative polarity in PSII. The 3D structure of the PSII complex (PDB code: 2axt). The protein atoms are colored according to their local polarity: red, white, and blue colors correspond to low, intermediate, and high values of polarity, respectively. The polarity degree was calculated as the number of polar groups of protein side chains and hetero atoms localized within the sphere of 7 Å radius divided by the total number of heavy atoms within the sphere. Redox cofactors are colored yellow. Abbreviations: PQ_A_ and PQ_B_, primary and secondary plastoquinones; Phe_D1_ and Phe_D2_, pheophytin molecules associated with the protein subunits D1 and D2 of PSII; P_680_, special pair of chlorophyll molecules; Y_Z_, redox-active tyrosine; Mn_4_CaO_5_, water-oxidizing manganese cluster. Panel (**b**): Polarity profile averaged for the structure shown in (**a**). The polarity profile marked by black circles along the ‘‘distance’’ axis correlates with the 3D structure shown in Figure 7a. Figure 7 is adapted from [109], with permission.

Figure 7 shows the distribution of polarity in the 3D structure of PSII. The polarity profile on the donor side of PS II is rather heterogeneous. There are two low-polar domains in the generally high-polar donor side of PS II. One of them is located in the vicinity of the Mn cluster; the others are closer to the protein–water boundary. 

### 4.2. EPR Measurements of ΔpH in Chloroplasts

Summarizing the results on ΔpH measurements in chloroplasts based on the use of the EPR techniques (spin probes, kinetics of pH-dependent electron transport), we conclude that the methods described above provide adequate measurements of the *intra*-thylakoid pH_in_ established in different metabolic states of chloroplasts. The results of the ΔpH estimations in isolated bean chloroplasts via two different methods (the use of pH-sensitive spin probes and the kinetic method) led us to virtually the same values of ΔpH as measured in the state of photosynthetic control, when the ATP synthase complexes were inactive (ΔpH ≥ 2.0–2.5 at pH_out_ ≈ 7.8–8.0). Under the photophosphorylation conditions, when the operation of the ATP synthase accelerates the efflux of protons from the lumen, ΔpH decreased (ΔpH ≈ 1.0–1.8) due to the proton drain from the lumen to the stroma. In this state, pH_in_ values usually did not drop below pH_in_ ≈ 6.0–6.2, facilitating ATP synthesis from ADP and orthophosphate P_i_ [34,88,91,102].

Our studies of ΔpH generation based on the use of EPR techniques led us to a re-examination of the problem of the lateral heterogeneity of ΔpH_in_ in thylakoid membranes [102]. Note that correct measurements of pH_in_ in different domains of chloroplasts are complicated by the nonuniform partitioning of the protons pumped into the lumen of granal (stacked) and stroma-exposed thylakoids. When we compared the results of ΔpH estimations in isolated bean chloroplasts obtained with pH-sensitive spin probes and from the post-illumination kinetics of P_700_^+^ reduction, we found that both methods led to virtually the same values of ΔpH, as measured in the state of photosynthetic control, when the ATP synthase complexes were inactive. Under the photophosphorylation conditions, ΔpH decreased due to the proton drain from the lumen to the stroma via the active ATP synthase complexes. In this state, however, ΔpH values derived from kinetic data were smaller than ΔpH measured with the pH-probing amines [88,89]. Such a discrepancy has been explained by the coexistence of thylakoids with different pH_in_ established in the granal and stromal thylakoids. This is because the kinetic method provides the “local pH-meter” technique, which is sensitive to a less significant decrease in pH_in_ inside the stroma-exposed thylakoids enriched with the ATP synthase complexes (Figure 5a). Otherwise, pH-indicating spin probes localized inside the thylakoids of both types give information on pH_in_ values averaged out at both the granal and stromal thylakoids (Figure 5b).

## 5. Future Directions

### 5.1. Fundamental Studies

The electrometrical method previously used for the study of Δψ generation in bRC from purple bacteria, PSI from spinach and cyanobacteria, and PSII from spinach can be extended for other photosynthetic samples, including RCs from green photosynthetic bacteria, heliobacteria, and PSI/PSII complexes from cyanobacteria and algae living under extreme conditions such as drought, high temperatures, and high light. Adaptation of photosynthetic organisms to extreme conditions can be reflected in changes in the kinetics and thermodynamics of electron transfer, as well as in the mutual arrangement of cofactors and protein dielectric properties. 

Recently, the effects of natural bioprotector disaccharide trehalose on the kinetics of electron transfer in PSI, PSII, and bRC have been studied in dry trehalose matrix and in solution (reviewed in [110]). It was shown that the desiccation in the trehalose matrix retarded the kinetics of forward electron transfer and significantly stabilized the pigment–protein complexes at room temperature by hindering diffusion, modifying the system of hydrogen bonds, and fixing the proteins in optimal conformational states. However, the effect of trehalose on the kinetics of Δψ generation was not studied so far. Such experiments may shed light on the mechanisms of the protein functioning under restricted conformational mobility and on the mechanism of trehalose’s exceptional protective effects. 

Correct measurements of ΔpH in chloroplasts are complicated by the existence of membrane-bound proton acidic groups [111,112,113,114,115], pH probes binding to membranes, and osmolarity effects [116,117,118], as well as by the uneven partitioning of the protons pumped into the lumen of the granal (stacked) and stroma-exposed thylakoids [102]. Such a discrepancy can be explained by the coexistence of thylakoids with different pH_in_, established in the lumen of the granal- and stroma-exposed thylakoids. We hope that future studies of ΔpH generation in chloroplasts will help solve the problem of lateral heterogeneity of the proton distribution in chloroplasts. Probably, the advances in high-resolution fluorescence confocal microscopy and the use of pH-sensitive fluorescence proteins [119,120,121,122] might help solve the above task in future work. It is noteworthy that the uneven lateral pH profile along the p-side of mitochondrial cristae in situ has been determined with fluorescent pH-sensitive green fluorescent protein (GFP) attached to OXPHOS complex IV in respiring HeLa cells [121]. The authors reported that the local pH at F_0_F_1_ dimers was 0.3 pH units less acidic than in the vicinity of complex IV. 

Another urgent task of membrane bioenergetics concerns the nature of the driving force used for the operation of the ATP synthase. According to the orthodox Peter Mitchell’s point of view, only delocalized proton gradients (not constrained to membrane domains) are required for proton-driven ATP formation [1,2]. An alternative concept implies the participation of the membrane-localized protons, because the essential portion of hydrogen ions consumed by chloroplasts is constrained to sequestered domains within the thylakoids [111,112,113,114,115]. This might necessitate a modification of Mitchel’s proposal concerning the bulk-to-bulk *pmf* difference. We hope that future structural studies, based on the use of high-resolution cryo-EM techniques [19,123], will help clarify this problem.

### 5.2. Practical Implications

The future perspectives on the practical implications of light-induced membrane potential generation measurements lie in the further development of the electrometrical methods and in the search for the most appropriate systems, which could provide high voltage for long time periods at room temperature. 

The photoelectrochemistry of PSII and PSI was previously studied mostly by measuring the photocurrent using a three-electrode mode set up by applying an external potential within the range of the open-circuit potential of the RC [124,125,126,127]. 

Recently, the stable Δψ generation by PSI, PSII, and bacterial chromatophores in the presence of trehalose has been measured using the modification of the direct electrometrical method (see [128] and references therein). This new approach was based on the use of the nitrocellulose membrane porous filters impregnated with pigment–protein complexes and sandwiched between two semiconductive indium tin oxide-coated transparent glass slides. In the presence of trehalose, a stable photovoltage was observed under steady-state illumination for about an hour at room temperature. The signal retained for approximately 24 h until the filter dried and could be mostly restored upon the addition of fresh buffer and redox mediators after one month of storage at room temperature. The data obtained provided a functional basis for the conversion of light energy into electricity upon prolonged light irradiation. The immersion of PSI, PSII, and chromatophores into a nitrocellulose membrane filter in the presence of a natural bioprotector—disaccharide trehalose—opens a prospective strategy for preventing their aggregation and denaturation over time. This approach can be used as a starting point for creating highly efficient and environmentally friendly devices for the stable conversion of light energy into electrical form. The energy role of the protons constrained to sequestered domains within the thylakoids needs future developments of the methods described above.

## 6. Conclusions

The consideration of biophysical methods of Δψ and ΔpH measurements based on the direct electrometry and EPR spectroscopy and the main results obtained by the study of different photosynthetic systems demonstrate that these methods remain relevant at present, and that they may have important fundamental and practical implications in the future. Such fundamental studies may clarify the molecular mechanisms of Δψ and ΔpH generation by the photosynthetic membrane proteins and intracellular organelles of organisms dwelling under extreme conditions of draught, high salinity, high temperature, and intense sunlight irradiance. The future practical application lays in the fact that the results obtained will be used for the creation of highly efficient and environmentally friendly solar-to-electricity converters capable of long-term functioning under physiological conditions. 

The experimental approaches described in this article provide the means for numerical determinations of both components of the protonmotive force, the electric potential, and the transmembrane pH difference, generated in different systems (biological and model membranes). We have illustrated these points by examples mainly borrowed from our previous publications. The advantages of using these methods are that they allow us to measure the Δψ and ΔpH values at different levels of the structural and functional organization of the membrane systems (membrane vesicles and energy-transducing organelles). One of the reasons for the scattering of ΔpH estimates could be the structural and functional variability of photosynthetic apparatus. The consideration of the results of ΔpH measurements based on different experimental methods (the noninvasive “kinetic” method and the use of pH-dependent spin probes), and their comparison with theoretical studies, allows us to make conclusions about the lateral heterogeneity of the ΔpH profile along the thylakoid membranes.
